# miR-153-3p via PIK3R1 Is Involved in Cigarette Smoke-Induced Neurotoxicity in the Brain

**DOI:** 10.3390/toxics11120969

**Published:** 2023-11-30

**Authors:** Qian Sun, Hailan Wang, Mingxue Yang, Haibo Xia, Yao Wu, Qizhan Liu, Huanwen Tang

**Affiliations:** 1 Dongguan Key Laboratory of Environmental Medicine, School of Public Health, Guangdong Medical University, Dongguan 523808, China; gwsunqian@gdmu.edu.cn (Q.S.); mxyang97@163.com (M.Y.); Wwyao0123@163.com (Y.W.); 2 Key Laboratory of Modern Toxicology of Shenzhen, Shenzhen Medical Key Discipline of Health Toxicology (2020–2024), Shenzhen Center for Disease Control and Prevention, Shenzhen 518055, China; 3 Center for Global Health, The Key Laboratory of Modern Toxicology, Ministry of Education, School of Public Health, Nanjing Medical University, Nanjing 211166, China; wanghailan9531@163.com (H.W.); haiboxia346@163.com (H.X.)

**Keywords:** cigarette, neurotoxicity, insulin resistance, miR-153

## Abstract

Cigarettes contain various chemicals that cause damage to nerve cells. Exposure to cigarette smoke (CS) causes insulin resistance (IR) in nerve cells. However, the mechanisms for a disorder in the cigarette-induced insulin signaling pathway and in neurotoxicity remain unclear. Therefore, we evaluated, by a series of pathology analyses and behavioral tests, the neurotoxic effects of chronic exposure to CS on C57BL/6 mice. Mice exposed to CS with more than 200 mg/m^3^ total particulate matter (TPM) exhibited memory deficits and cognitive impairment. Pathological staining of paraffin sections of mouse brain tissue revealed that CS-exposed mice had, in the brain, neuronal damage characterized by thinner pyramidal and granular cell layers and fewer neurons. Further, the exposure of SH-SY5Y cells to cigarette smoke extract (CSE) resulted in diminished insulin sensitivity and reduced glucose uptake in a dose-dependent fashion. The PI3K/GSK3 insulin signaling pathway is particularly relevant to neurotoxicity. microRNAs are involved in the PI3K/GSK3β/p-Tau pathway, and we found that cigarette exposure activates miR-153-3p, decreases PI3K regulatory subunits PIK3R1, and induces Tau hyperphosphorylation. Exposure to an miR-153 inhibitor or to a PI3K inhibitor alleviated the reduced insulin sensitivity caused by CS. Therefore, our results indicate that miR-153-3p, via PIK3R1, causes insulin resistance in the brain, and is involved in CS-induced neurotoxicity.

## 1. Introduction

Cigarette smoking is a well-established risk factor for various neurodegenerative diseases, such as Alzheimer’s disease (AD), Parkinson’s disease (PD), and multiple sclerosis. Recent studies have suggested a link between smoking and insulin resistance in the brain, which may contribute to the development of these diseases [1,2]. 

There is an epidemiological and mechanistic link between cigarette smoking and insulin resistance. According to the 2014 Atlanta Surgeon General’s Report, the risk of Type 2 diabetes (T2D) is 30–40% higher among smokers than for nonsmokers. A study conducted in Shanghai, China, followed 50,000 men for an average of 5.4 years. In this study, those who smoked more than 20 cigarettes per day had an elevated hazard ratio of incident insulin resistance of 1.25 (95% CI: 1.00, 1.56), and men with a smoking history of ≥40 pack-years had an elevated hazard of insulin resistance of 1.28 (95% CI: 1.04, 1.57) [3].

Insulin, a pancreatic peptide that is involved in the regulation of glucose metabolism, has a multifaceted role in the central nervous system (CNS). Insulin resistance in the brain is a condition in which the cells become less responsive to the effects of insulin, leading to a decline in cognitive function and an increased risk of neurodegeneration [4]. Therefore, AD could be a brain-specific form of diabetes, namely type 3 diabetes [5,6,7]. On a molecular level, insulin insensitivity in the periphery or in the CNS can reflect a wide range of underlying changes, including reduced insulin receptor concentrations, a reduced binding affinity of insulin, and the disruption of the insulin signaling cascade [8].

In brain insulin resistance and neurodegeneration, the PI3K/AKT/GSK3β pathway may be involved in signal transduction. Insulin resistance decreases PI3K/AKT/GSK3β activity [9]. In peripheral tissues, reduced PI3K activity inhibits the expression and translocation of GLUT4 [10,11], a glucose transporter protein. Moreover, reduced PI3K activity leads to Tau protein hyperphosphorylation in the CNS [12,13]. This hyperphosphorylation is believed to be a pathogenic mechanism for AD [14]. Mice over-expressing GSK-3β in the forebrain exhibit Tau hyperphosphorylation in an AD-relevant epitope [15]. Relative to non-smokers, smokers have a higher incidence of insulin resistance in the brain, possibly due to the toxic effects of the chemicals in cigarette smoke (CS) [16].

Currently, miRNAs are considered potential pathogenic factors due to their mode of functioning and pleiotropic mechanism of action. They are now serving as biomarkers for various common diseases, including neurodegeneration and AD [17,18]. In AD patients, Hsa-miR-485-3p is over-expressed; its inhibition slows the progression of the disease [19]. Hsa-miR-103a-3p is related to cognitive impairment in PD [20], and miR-29a and miR-29b serve as biomarkers for Alzheimer’s disease [21]. Although the mechanisms by which cigarettes cause insulin resistance in peripheral tissues have been extensively studied [22,23,24,25], there are few studies on the mechanisms by which cigarettes promote the development of neurodegenerative diseases by causing insulin resistance in the brain. Therefore, identifying miRNAs associated with the brain insulin signaling pathway as a potential mechanism for developing and progressing cigarette neurotoxicity could open a new avenue for therapeutic options. 

## 2. Materials and Methods 

### 2.1. Animal Subjects

For our study, we sourced six-month-old male C57BL/6N mice from the Guangdong Medical Laboratory Animal Center, located in Guangdong, China. Throughout the study, we ensured the humane treatment of these mice. Our experimental procedures adhered strictly to the guidelines set forth by Institutional Animal Care and aligned with the National Research Council’s recommendations for laboratory animal care. The Ethics Committee at the Shenzhen Center for Disease Control and Prevention (SZCDC) granted approval for the use of these animals in our research. We housed the mice in the Animal Lab Center Building of the SZCDC, providing them with unrestricted access to water and a standard rodent diet. The environmental conditions were carefully controlled, maintaining a consistent 12 h light/dark cycle and a temperature of 22 ± 2 °C.

In our study, the male C57BL/6N mice were systematically allocated into four distinct groups (*n* = 16 for each group). The primary objective was to assess the neurotoxic effects of cigarette smoke (CS). For this purpose, we utilized 1R6F research cigarettes, sourced from the University of Kentucky’s Center for Tobacco Reference Products in the USA. The exposure protocol involved subjecting the mice to varying concentrations of total particulate matter (TPM) from CS—specifically, 0, 100, 200, or 300 mg/m^3^. Referring to relevant studies for conversion, the exposure dose of 100, 200, 300 mg/m^3^ in mice is equivalent to mild (0–0.5 pack/day), moderate (0.5–1 pack/day), and severe smoking (>1 pack/day) in humans [26,27].

This exposure occurred in a controlled environment for 60 min, twice daily with a 4 h interval, and was repeated five days a week over a six-month period. The exposure was facilitated using a whole-body system provided by Huironghe Technology CO., Ltd., Beijing, China. During the exposure sessions, we continuously monitored and recorded the chamber’s humidity, temperature, and oxygen levels.

Following the exposure period, we conducted two behavioral tests on the mice, now aged 12 months, to evaluate their learning, memory, and cognitive functions. The final step involved the careful dissection of the hippocampi for subsequent detailed analyses. All investigators were blinded to treatment groups.

### 2.2. Assessment of Behavior

In our behavioral assessment, we employed two specific tests for the mice: the Morris Water Maze (MWM) and the Novel Object Recognition (NOR) tests. We conducted all behavioral evaluations in a controlled environment, specifically in a laboratory with regulated air and soundproofing, during the hours of 09:00 to 17:00. To ensure the integrity of each test and to eliminate any residual olfactory cues that could influence the behavior of the mice, we meticulously cleaned the testing equipment with a solution of 70% ethanol and water after each session.

#### 2.2.1. The Novel Object Recognition (NOR) Test Procedure

The Novel Object Recognition (NOR) test is divided into three distinct phases: habituation, training, and the actual test. During the training phase, each mouse was allowed to familiarize itself with two yellow rectangular objects. On the day of the test, a variation was introduced by replacing one of the yellow rectangles with a green cylinder. We then observed the mice for 5 min as they freely moved between the two objects. To quantify their ability to recognize the new object, we calculated a Discrimination Index (DI) for each group. This index measures the difference in time spent exploring each object relative to the total exploration time. Mathematically, it is represented as DI = (T1 − T2)/(T1 + T2), where T1 and T2 are the times spent exploring the first and second objects, respectively. A DI value of 0 indicates no preference or distinction between the two objects.

#### 2.2.2. Conducting the Morris Water Maze (MWM) Test

The MWM test was used to evaluate spatial and long-term memory according to a previous method [28]. The test involved a large circular pool, measuring 150 cm in diameter and 50 cm in height, which was filled with a non-transparent liquid created by mixing water with milk to achieve a white color. At the center of the pool, a platform with a diameter of 10 cm was positioned just 1 cm below the water surface. For monitoring and analysis, a camera linked to specialized computer software was set up (ANY-maze software V2.0). 

The pool was divided into four equal quadrants (I–IV), and the system was capable of automatically tracking various parameters such as the path length of the mice, their escape latency, and the time they spent in each quadrant. During the training phase, the mice underwent four trials each day, each lasting 60 s, over a period of five days. These trials were designed to train them to locate a visibly placed platform. If a mouse failed to find the platform within the allotted time, it was gently guided to it. A probe test was conducted six days after the completion of these navigation trials. In this test, the platform was removed from the pool, and the mice were introduced into the water from the quadrant directly opposite the quadrant in which the platform was located. They were then given 2 min to search for the now-absent escape platform.

### 2.3. Hematoxylin and Eosin (H&E) Staining

To assess the extent of cerebral injury, we conducted detailed histological analyses of the mouse brain tissue. Initially, the brain samples were embedded in paraffin and sectioned into slices with a thickness of 4 μm. Following this, the sections underwent a thorough dewaxing process, involving three 5 min immersions in xylene. This was succeeded by a rehydration sequence using a descending ethanol series (100%, 95%, 85%, and 75%) for 5 min at each concentration, and then the sections were washed thrice in PBS, also for 5 min each. For nuclear visualization, we stained the sections with hematoxylin for 5 min, followed by a brief rinse under tap water for 1 min. Subsequently, the sections were counterstained with eosin for a duration ranging from 30 s to 1 min. Photographs of the samples were captured using an Olympus BX60 light microscope, which was integrated with a high-resolution digital camera, originating from Japan. The scoring of the pathological sections was conducted using a double-blind method.

### 2.4. Nissl Staining

Nissl staining, a technique employed for highlighting Nissl bodies within the neuronal cytoplasm, was utilized in our study. The process began with dehydrating the sections, followed by clearing them in xylene and then mounting them using a non-fluorescent medium. For the staining, the slides were immersed in a Nissl Staining Solution provided by Sangon Biotech, China, and maintained at room temperature for 30 min. Subsequent to the incubation, the slides were washed twice using distilled water. This staining procedure resulted in the Nissl bodies appearing to have a distinctive purple-blue hue. To quantitatively analyze the histochemical intensity of the neuronal cells, particularly in the CA1 region of the hippocampus, we employed ImageJ 2022 software, developed by the National Institutes of Health in Bethesda, MD, USA. The analysis of the images was conducted by two researchers who were unaware of the group allocation, and each group randomly analyzed images of six mice. For brain slices, each slice was analyzed by selecting the same partition.

### 2.5. Neuron-Specific Nuclear Protein (NeuN) Immunohistochemistry

In brief, brain sections were washed thrice with PBS and then incubated in a hydrogen peroxide block for 10 min to eliminate endogenous peroxidase activity. This was followed by a 60 min room temperature incubation with a protein blocking agent. Subsequently, the sections were incubated overnight at 4 °C with a primary antibody, Anti-NeuN (Beyotime Biotechnology, Shanghai, China), at a dilution of 1:50. The next day, the sections were incubated for 60 min with biotinylated goat anti-polyvalent enzyme, followed by a 30 min incubation with streptavidin peroxidase. The signal was visualized using a Diaminobenzidine (DAB)-hydrogen peroxide substrate, resulting in a brown coloration. Nuclei were stained for 3 min with a hematoxylin solution (1 mg/mL, Sigma, St. Louis, MO, USA, diluted 1:3000), yielding a blue color. The quantification of NeuN-positive neurons was performed in a double-blind manner using ImageJ 2022 software.

To effectively compare differences between groups and mitigate individual variability within groups, we established a standard reference value. The result from the first mouse in the 0 mg/m^3^ group was designated ‘WT’. All other sections’ NeuN-positive neuron quantification results were then normalized to this baseline value. The analysis of the images was conducted by two researchers who were unaware of the group allocation, and each group randomly analyzed images of six mice. For brain slices, each slice was analyzed by selecting the same partition.

### 2.6. Preparation of Cigarette Smoke Extract (CSE) 

CSE was prepared as previously reported [23]. In summary, smoke from a 1R6F research cigarette (sourced from the University of Kentucky, Lexington, KY, USA) was directed into a flask containing 10 mL of MEM medium at 37 °C. This process was facilitated by a vacuum pump (model EVP 2XZ-2C, from Hangzhou, China) operating at a steady flow rate. Post-production, the CSE solution underwent sterilization through a 0.22 µm pore filter (manufactured by Schleicher & Schuell GmbH, Dassel, Germany) and its pH was adjusted to 7.4. For quality assurance, absorbance readings at 320 nm and 540 nm were taken and normalized. The CSE was deemed suitable for use if the difference in optical density (ΔOD) between these two readings (A320–A540) fell within the range of 0.9 to 1.2. The freshly prepared solution, defined as 100% CSE, was then diluted with the medium and used in experiments within an hour of its preparation.

### 2.7. Cell Culture and Treatment

SH-SY5Y human neuroblastoma cells were acquired from the Shanghai Institute of Cell Biology under the Chinese Academy of Sciences. These cells were propagated in MEM medium supplied by Thermo Fisher Scientific, Waltham, MA, USA, which was enriched with 15% fetal bovine serum (FBS) and 2 mM L-glutamine from Sigma Aldrich, St. Louis, MO, USA, along with 100 mg/mL streptomycin and 100 U/mL penicillin, both from Thermo Fisher Scientific. The culture conditions were maintained at 37 °C in a humidified environment with a 5% CO_2_ atmosphere. Upon reaching a confluence of 70–80%, the SH-SY5Y cells were rinsed with PBS. Subsequently, they were subjected to varying concentrations of CSE (0, 1, 2, 4, 8% or 16%) for periods of either 24 or 48 h.

### 2.8. MTT (3-(4,5-Dimethylthiazol-2-yl)-2,5-diphenyltetrazolium bromide) Assay

Cell viability inhibition was assessed using the MTT assay. SH-SY5Y cells were seeded in a 96-well plate at a density of approximately 10,000 cells per well. After cell adherence, the original medium was replaced with media containing various concentrations of CSE to establish control groups (untreated) and several experimental groups (treated with different concentrations of CSE). Following a predetermined incubation period (e.g., 24 and 48 h), the CSE-containing medium was removed. MTT solution, typically 10% of the culture medium volume, was then added to each well and incubated for 3–4 h. The MTT solution was subsequently discarded, and an appropriate amount of DMSO was added to dissolve the formazan crystals formed. Absorbance was measured at a wavelength of 570 nm.

### 2.9. Glucose Uptake Assay 

Glucose uptake assays were performed using Glucose Uptake Assay kits (Abcam, Waltham, MA, USA). After exposure to CSE, SH-SY5Y cells were treated with 200 nM insulin for 20 min to activate the glucose transporter and washed with PBS. After the addition of 2-deoxyglucose, they were incubated for 20 min at 37 °C. The cells were washed with PBS. Freeze/thaw the cells once. The cells were heated at 85 °C for 40 min, and placed on ice to cool down. Begin by chilling the cell lysates on ice for a duration of 5 min. Subsequently, neutralization is achieved by incorporating 10 µL of Neutralization Buffer. Prepare a sufficient quantity of reagents, taking into account the total number of assays planned, including controls, samples, and standards. For the plate reading process, incubate the plate at a temperature of 37 °C for 40 min, ensuring it is shielded from light exposure. The fluorescence should then be measured with an excitation/emission wavelength of 535/587 nm.

### 2.10. RNA Extraction and Quantitative Real-Time Polymerase Chain Reaction (RT-PCR) 

Total RNA was meticulously extracted from SH-SY5Y cells using the Trizol reagent (Invitrogen, Carlsbad, CA, USA). This was followed by further purification of the RNA with the miRNeasy Mini Kit (Qiagen, Dusseldorf, Germany). Reverse transcription was then carried out using the miScript II RT Kit (Qiagen, Dusseldorf, Germany), adhering closely to the manufacturer’s instructions. For the quantification of miR-153 and other predicted miRNAs, which are conserved across human and murine cells, quantitative real-time PCR (qPCR) was performed. This qPCR process employed the miScript SYBR Green PCR Kit (Qiagen, Dusseldorf, Germany). Amplification and detection of the qPCR products were executed using the Mastercycler ep realplex system (Eppendorf, NY, USA). Primer sequences for these miRNAs, along with U6 snRNA, chosen as the internal control for normalization, are detailed in Table 1. This approach ensures accurate normalization and enables a comprehensive comparative analysis of the expression levels of miR-153 and the other predicted miRNAs across various samples.

### 2.11. Cell Transfection

In six-well plates, we seeded 5 × 10^5^ SH-SY5Y cells, allowing them to grow until they reached 80% confluence. These cells were then subjected to transfection using either a human miR-153 mimic or inhibitor, or a non-targeting scramble control, all of which were sourced from Ribobio in Guangzhou, China. Both the mimic and the inhibitor targeted has-miR-153-3p (MIMAT0000439:5′UUGCAUAGUCACAAAAGUGAUC). SH-SY5Y cells, at a concentration of 2 µg/well, were transfected using Lipofectamine™ 2000 (Invitrogen) according to the manufacturer’s protocol. The miR-153 mimic or the inhibitor, at a final concentration of 100 nM, was added to control cells or to CSE-exposed cells. All assays were performed in triplicate. After transfection, the cells were treated with 200 nM insulin for 10 min, and then harvested for total RNA and protein extraction or for miRNA isolation. The PI3K inhibitor LY294002 (20 μM) (Selleck, Shanghai, China) was used according to a standard protocol.

### 2.12. Protein Extraction and Western Blot Assays 

Hippocampal tissue from mice and SH-SY5Y cell samples were harvested and subsequently lysed utilizing RIPA protein extraction reagent, procured from Beyotime in Beijing, China. To this lysis buffer, we added both a protease inhibitor cocktail and a phosphatase inhibitor cocktail, each obtained from Roche, Basel, Switzerland. The quantification of protein concentrations was carried out employing the BCA protein assay, following the protocol provided by the Beyotime Institute of Biotechnology in Shanghai, China.

Equal amounts of protein samples (20 µg) were separated by electrophoresis using 10% SDS-PAGE gels, and proteins were transferred to polyvinylidene fluoride (PVDF) membranes (Millipore, Burlington, MA, USA). The membranes were incubated overnight at 4 °C with the appropriate primary antibodies against GAPDH (1:1000 dilution Sigma, St. Louis, MO, USA); PI3K/p110 or PI3K/p85 (1:1000 dilution, Cell Signaling Technology, Boston, MA, USA); GSK3β (1: 250 dilution, Thermo Fisher Scientific, Waltham, MA, USA); or p-Tau S202 T205 (1:1000 dilution, Thermo Fisher Scientific, Waltham, MA, USA). After 1 h of antibody exposure, samples were incubated with an appropriate horseradish peroxidase-conjugated secondary antibody for an additional 1 h (Cell Signaling Technology, Boston, MA, USA). Blots were quantified using densitometry and normalized using GAPDH or Total Tau. For densitometric analyses, protein bands on the blots were measured using ImageJ 2022 software.

### 2.13. Statistical Analysis

Statistical evaluations were performed utilizing GraphPad Prism 8, a product of GraphPad Prism 9 Software based in La Jolla, CA, USA. Data were expressed as mean values ± standard deviation (SD). To assess the statistical disparities among different treatment groups, we employed a one-way ANOVA, followed by Tukey’s post hoc test for multiple comparison purposes. A *p*-value of less than 0.05 was set as the threshold for statistical significance.

## 3. Results

### 3.1. Cigarette Smoke (CS) Impairs Learning Memory and Cognitive Abilities in Mice

**Figure 1:**
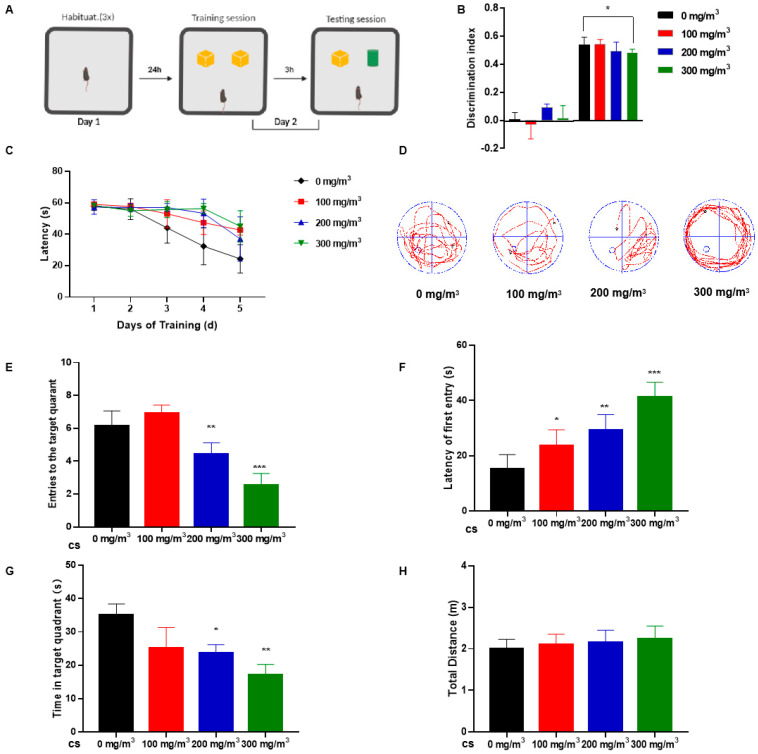
Cigarette smoke (CS) impairs learning memory and cognitive abilities of mice. C57BL/6 mice were exposed to 0, 100, 200, or 300 mg/m^3^ total particulate matter (TPM) CS for six months. After this exposure, two behavioral tests were performed. (1) The novel object recognition (NOR) test. (**A**) On the first day, the mice were habituated for three 5 min sessions, each 1.5 h apart. On the second day (training session), mice were placed in the center of the device and were free to explore two identical objects (yellow blocks) for 5 min. After 3 h (test session), one object was replaced with an object of a different color and shape (green cylinder). Mice explored objects freely for 5 min. (**B**) Histograms representing the discrimination index (DI) for each experimental group during training (left) and testing (right). Calculation methods are in the Experimental Methods section. (2) The Morris water maze (MWM) test. (**C**) Differences in latency of platform seeking for mice exposed to various concentrations of CS during a five-day training period. After removing the platform, probe test trials were conducted six days after the training session. (**D**) The swimming trajectories of mice during the probe test. (**E**) Numbers of entries into the target quadrant. (**F**) Latency of first entry to the target quadrant. (**G**) Time spent in the target quadrant. (**H**) The total distance travelled in the platform quadrant in the probe trial of the MWM test. Bar graphs show SD (*n* = 16 for each group), * *p* < 0.05, ** *p* < 0.01, *** *p* < 0.001 vs. 0 mg/m^3^ group.

Exposure to CS did not affect food or water intake by mice (Supplementary Figure S1A,B). Although weights of the groups did not differ statistically, there was a tendency for body weight to decrease with increasing CS concentrations (Supplementary Figure S1C). The NOR test assessed the short-term memory and learning of mice [29]. The task relied upon the tendency of rodents to attend to a novel object more than to a familiar one. During the training session, mice spent similar times exploring each object. In contrast, during the testing session, CS-exposed groups of mice exhibited lower DI as assessed at 3 h after training, suggesting that they had trouble discerning the difference between the two objects (Figure 1A,B). 

During the training period for the MWM test, CS-exposed mice exhibited poor learning ability and needed more time to search for a platform (Figure 1C). During the evaluation phase of the MWM test, the platform was removed. The outcomes indicated that mice subjected to cigarette smoke (CS) exhibited a prolonged duration in locating the former platform site, as illustrated in Figure 1D. We quantified the frequency with which mice traversed the area where the platform was previously located. Key parameters such as the number of times mice entered the target quadrant, the duration spent within this quadrant, and the overall distance navigated in the vicinity of the platform quadrant were meticulously recorded. These measures aimed to discern any variances among groups subjected to different levels of CS exposure. Notably, CS-exposed mice demonstrated a delayed response in identifying the target quadrant, a reduced frequency of platform crossings, and diminished time spent in the relevant quadrant, as depicted in Figure 1E–G. There was no difference in the total distance traveled by the mice in the water maze, which suggests to some extent that cigarette smoke did not affect the mice’s motor abilities (Figure 1H).

These results indicate that CS-treated mice exhibit impaired memory and cognitive performance; however, behavioral tests revealed no appreciable impairment of motor ability.

### 3.2. CS Induces Pyramidal Cell Damage in the CA1 Region of the Hippocampus and Histopathological Changes in the Hippocampi of Mice

**Figure 2:**
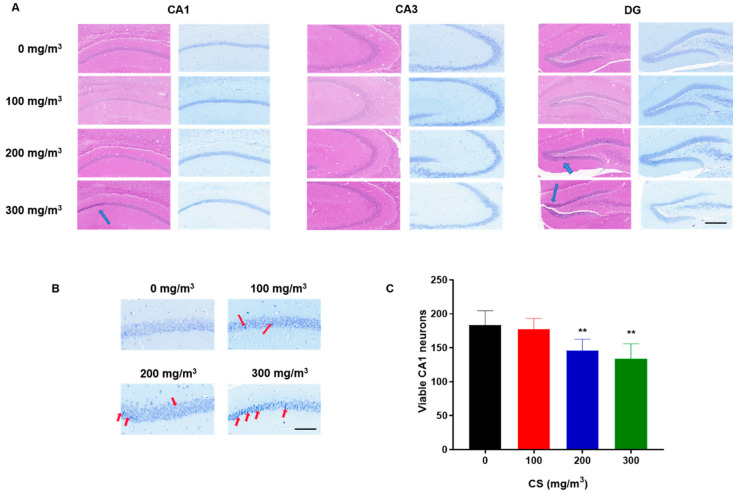
CS induces pyramidal cell damage in the CA1 region and histopathological changes in the hippocampus of mice. C57BL/6 mice at 6 months of age were exposed to 0, 100, 200, or 300 mg/m^3^ TPM CS for 6 months. (**A**) Representative photomicrograph of H&E staining (left) and Nissl staining (right) in the DG, CA3, and CA1 regions of the mouse hippocampus. Scale bar = 200 μm. The blue arrows mark the locations of the apparent contractions of the pyramidal cells. (**B**) For the CS-exposed group, Nissl staining showed a consolidation of the pyramidal cell layer in the CA1 region of the hippocampus, and a decrease in the number of neurons with normal structure. Arrows indicate the dark, shrunken, and damaged neurons. Scale bars = 50 μm. (**C**) Cell densities were represented on the graphs by counting surviving cells per field under a light microscope. With a dose-effect relationship, CS exposure resulted in a reduction in the number of pyramidal neurons in the CA1 region of the mouse hippocampus. All data are presented as means ± SD for experiments conducted in triplicate. ** *p <* 0.01 vs. 0 mg/m^3^ group (*n* = 6 for each group).

Histopathological alterations were evident in the H&E-stained hippocampal sections. In contrast to the control group, where cells appeared healthy and intact, the hippocampi of animals exposed to cigarette smoke (CS) displayed a significant presence of pyknotic cells. These changes were predominantly observed in the pyramidal cell layer of the CA1 region and the dentate gyrus (DG), as highlighted by blue arrows in Figure 2A. The overall structure of the hippocampus is shown in Figure S2.

Nissl staining revealed that, in the control group, the Nissl bodies were large and numerous, indicating that protein synthesis was stronger in nerve cells, whereas the numbers of Nissl bodies in CS-treated groups were lower, and the staining was shallow and unclear (Figure 2). The numbers of neurons in the CA1 region decreased with increases in CS concentration (Figure 2C). However, the numbers of neurons in the CA3 and DG region were not observed to change (Figure S3). These results indicate that the exposure of mice to CS reduces the number of pyramidal neurons in the CA1 region of the hippocampus in a dose-effect relationship.

### 3.3. CS Causes Hippocampal Neuron Deficiency, Disruption of PI3K/GSK3β Insulin Signaling Pathways, and Tau Protein Hyperphosphorylation

**Figure 3:**
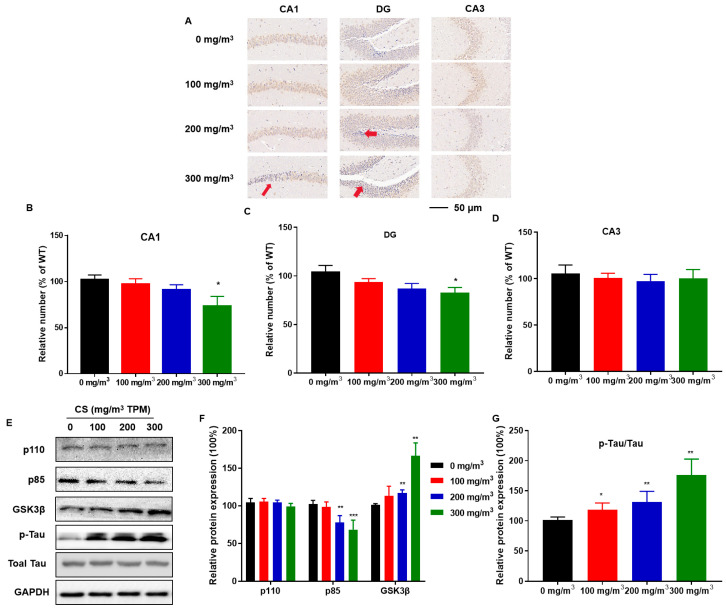
CS causes hippocampal neuron deficiency, disruption of PI3K/GSK3β insulin signaling pathways, and Tau protein hyperphosphorylation in mice. C57BL/6 mice at 6 months of age were exposed to 0, 100, 200, or 300 mg/m^3^ TPM CS for 6 months. Mouse hippocampi were stained for neuron-specific nuclear protein (NeuN) for assessment of the numbers of neurons in the DG, CA3, and CA1 regions (*n* = 6 for each group). (**A**) The distribution and number of NeuN-immunoreactive neurons in DG, CA3, and CA1 regions in the mouse hippocampus. Scale bars = 50 μm. Red arrows are representative phenotypes. (**B**–**D**) Mean percentages of NeuN immunoreactive neurons in the hippocampus. (**E**) Expressions of PI3K/GSK3β insulin signaling-related proteins and Tau protein were determined using Western blots (*n* = 3 for each group). (**F**,**G**) Relative protein levels of phosphoinositide 3-kinase (PI3K) catalytic subunits (p110), phosphoinositide-3-kinase regulatory subunits (p85), GSK3β, and p-Tau were measured. Blots were quantified using densitometry and normalized using GAPDH to correct for differences in the loading of proteins. All data are presented as means ± SD for experiments conducted in triplicate. * *p* < 0.05, ** *p* < 0.01, *** *p* < 0.001 versus the 0 mg/m^3^ group.

Hippocampal tissue sections were stained for the neuronal marker, NeuN, to observe the effect of CS on hippocampal neurons. The distribution and number of NeuN-immunoreactive neurons in the pyramidal cell layer of the CA1 region and the granular cell layer of the DG region were lower in the high-dose CS exposure group than in the control group (Figure 3A–C). Thus, the hippocampus is vulnerable to CS, which induces the pyramidal cell layer in the CA1 region to become thinner, decreasing the number of hippocampal neurons. 

To determine whether CS exposure causes disorders in insulin signaling, we investigated the relevant protein expression in the hippocampus. Compared to the 0 mg/m^3^ TPM CS-exposed group, the 200 and 300 mg/m^3^ TPM CS-exposed groups exhibited a decrease in phosphoinositide-3-kinase regulatory subunits (p85); the expression of phosphoinositide 3-kinase (PI3K) catalytic subunits (p110) did not change (Figure 3D,E). CS exposure increased GSK3β levels (Figure 3D,E). CS-exposed mice had a higher GSK3β-mediated hyperphosphorylation of two key Tau residues, Ser202 and Thr205 [30]. Tau protein phosphorylation increased with increasing CS exposure (Figure 3D,E). 

### 3.4. CSE Exposure Inhibits Cell Viability, Decreases Insulin Sensitivity, Induces Tau Hyperphosphorylation, and Affects the PI3K/GSK3β Signaling Pathway in SH-SY5Y Cells

**Figure 4:**
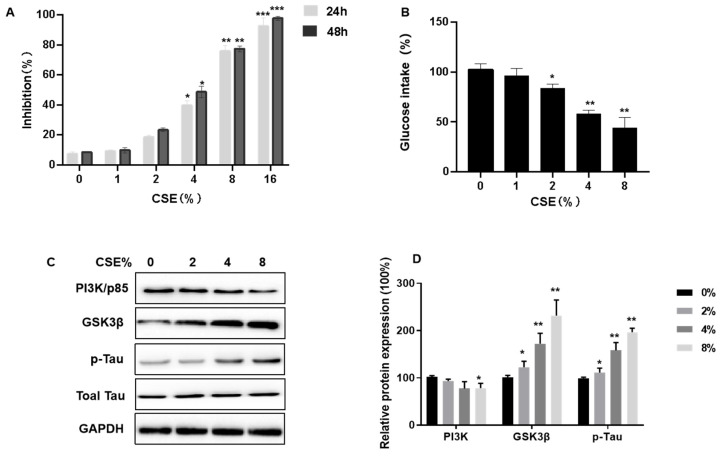
Cigarette smoke extract (CSE) inhibits cell viability, decreases insulin sensitivity, induces Tau hyperphosphorylation, and affects the PI3K/GSK3β signaling pathways in SH-SY5Y cells. SH-SY5Y cells were cultured in plates and exposed to various concentrations of CSE for 24 h or 48 h. Then, 200 nM insulin was added, and cells were treated for 20 min. (**A**) Growth inhibition was determined by MTT assays. (**B**) After being treated with various concentration of CSE for 48 h, the effect of CSE on cellular glucose uptake by SH-SY5Y cells was determined by Glucose Uptake Assay kits (*n* = 6 for each group). (**C**,**D**) Relative protein levels of PI3K (p85), GSK3β, and p-Tau were measured (*n* = 3 for each group). Blots were quantified by densitometry and normalized with the use of GAPDH to correct for differences in the loading of proteins. All data are presented as means ± SD for experiments conducted in triplicate. * *p* < 0.05, ** *p* < 0.01, *** *p* < 0.01 versus the 0% CSE group.

We conducted a study to evaluate the impact of cigarette smoke extract (CSE) on the viability and insulin sensitivity of SH-SY5Y neuronal cells. These cells were subjected to varying concentrations of CSE, ranging from 0 to 16%, for periods of either 24 or 48 h, to observe the effects on cell viability post-exposure. The results, ascertained through MTT assays, indicated a dose-dependent reduction in SH-SY5Y cell proliferation due to CSE, as depicted in Figure 4A.

Using Glucose Uptake Assay kits, we tested the possibility that CSE causes a dysfunction in insulin sensitivity. Cells were exposed to CSE (1, 2, 4, or 8% CSE) for 24 h, then 200 nM insulin was added for 10 min. Compared to controls, there was a lower glucose intake (Figure 4B). We measured the expression of related proteins, including PI3K/p85, GSK3β, and p-Tau/Total Tau, to determine how CSE impairs PI3K/GSK3β/Tau signaling pathways. CSE exposure decreased p85 expression (Figure 4C,D). As the CSE concentration increased, GSK3β expression increased, along with Tau hyperphosphorylation (Figure 4C,D). Thus, for SH-SY5Y cells, CSE exposure inhibits cell viability, decreases insulin sensitivity, induces Tau hyperphosphorylation, and affects the PI3K/GSK3β signaling pathway. 

### 3.5. miR-153-5p Expression Is Elevated by CSE and Targets the 3′UTR Site of PIK3R1

The PI3K regulatory subunit p85 has three common isomers; however, based on a database search (HPA RNA-seq normal tissues and Mouse ENCODE transcriptome data), only the alpha isomer (PIK3R1) is expressed in human and mouse brains.

**Figure 5:**
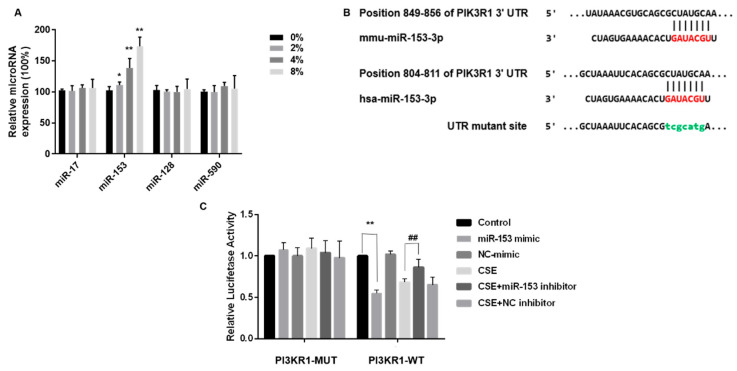
miR-153-5p expression is elevated by CSE and targets the 3′UTR site of PIK3R1. Bioinformatics prediction software Targetscan (https://www.targetscan.org) accessed on 1 March 2023, was used to predict binding sites for the phosphatidylinositol 3-kinase regulatory subunit alpha (PIK3R1). (**A**) Quantitative RT-PCR was used to validate miRNA combined with mRNA. (**B**) Schematic graph of the binding sites of miR-153-3p in the 3′UTR of PIK3R1 of mice and humans. SH-SY5Y cells were transfected with PIK3R1-MUT or PIK3R1-WT for 24 h, then co-transfected with 100 nM miR-153 mimic or negative control for 24 h. Cells were exposed to 0 or 4% CSE, then co-transfected with 100 nM miR-153 inhibitor or negative control for 24 h. Then, 200 nM insulin was added for 20 min. (**C**) Luciferase activity. * p < 0.05 and ** p < 0.01, different from control and 4% CSE. *## p* < 0.01, different from SH-SY5Y cells treated with CSE alone. All data are presented as means ± SD for experiments conducted in triplicate (*n* = 6 for each group).

TargetScan was employed to identify the potential miRNAs targeting PIK3R1. In the list of PIK3R1 predicted targets, miR-17-5p, miR-153-3p, miR-128-3p, and miR-590-5p were combined with PIK3R1 and were highly homologous to humans and mice. After CSE exposure of SH-SY5Y cells, only miR-153, one of the four target miRNAs, was elevated (Figure 5A). Thus, miR-153 was selected for more in-depth analysis. 

We determined whether miR-153 suppressed PIK3R1 by interacting with the PIK3R1 3′UTR. These luciferase reporter plasmids were co-transfected into SH-SY5Y cells with the wild-type PIK3R1 3′UTR plasmid or a MUT PIK3R1 plasmid. MUT PIK3R1 3′UTRs contained mutations at predicted miR-153 binding sites, TCGCATG (Figure 5B). SH-SY5Y cells were transfected with either NC or an miR-153 mimic. In line with the hypothesis of a direct interaction between miR-153 and the 3′UTR of PIK3R1, we observed a decrease in luciferase activity in SH-SY5Y cells transfected with the wild-type PIK3R1 3′UTR plasmid following miR-153 overexpression, as shown in Figure 5C (right panel). However, miR-153 overexpression did not affect PIK3R1 in cells transfected with mutated 3’ UTR plasmids (Figure 5C, left). 

Although CSE exposure also reduced the luciferase activity, transfection with an miR-153 inhibitor, followed by CSE exposure, restored luciferase activity, confirming that PIK3R1 is a target of miR-153 (Figure 5C). Thus, CSE elevates miR-153-5p expression and targets the PIK3R1 3′ UTR site.

### 3.6. miR-153-5p via PI3K Is Involved in CSE-Induced Brain Insulin Resistance and Tau Protein Hyperphosphorylation

**Figure 6:**
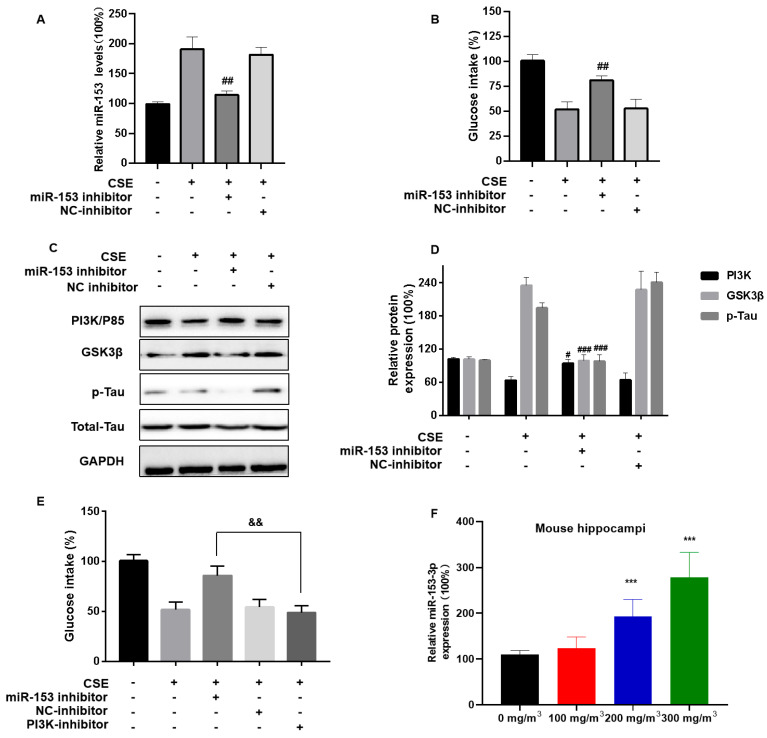
miR-153-5p via PI3K is involved in CSE-induced brain insulin resistance and Tau protein hyperphosphorylation. SH-SY5Y cells were transfected with miR-NC or an miR-153-3p inhibitor (100 nM) for 24 h, then incubated with 0 or 4% CSE for 24 h. Insulin (200 nM) was added for 20 min. (**A**) The levels of miR-153-3p were measured using quantitative RT-PCR. (**B**) Cellular glucose uptake was determined with Glucose Uptake Assay kits. (**C**) Western blots were performed, and (**D**) relative protein levels of PI3K (P85), GSK3β, and p-Tau were measured (*n* = 3 for each group). CSE-treated SH-SY5Y cells were transfected with NC-inhibitor, miR-153 inhibitor (100 nM) and/or PI3K inhibitor (50 nM) for 24 h, then incubated with 0 or 4% CSE for 24 h. (**E**) Cellular glucose uptake was determined using Glucose Uptake Assay kits. (**F**) Quantitative RT-PCR was used to assess the levels of miR-153-3p in mouse hippocampi (*n* = 6 for each group). *** *p* < 0.001, different from control mice. # *p* < 0.05, ## *p* < 0.01, ### *p* < 0.001 different from SH-SY5Y cells treated with CSE alone. && *p* < 0.01, different from CSE-exposed SH-SY5Y cells transfected with an miR-153-3p inhibitor. All data are presented as means ± SD for experiments conducted in triplicate.

To evaluate the effect of CSE exposure and miR-153 on brain insulin resistance and neuronal damage, SH-SY5Y cells were transfected with an miR-153 inhibitor, then exposed to CSE. The miR-153 inhibitor restored the glucose intake levels (Figure 6A,B). The inhibitor also ameliorated the CSE-induced p85 level downregulation and Tau protein hyperphosphorylation (Figure 6C,D). SH-SY5Y cells were transfected with the miR-153 inhibitor and/or the PI3K inhibitor LY294002 for 24 h, then incubated with 4% CSE for 24 h. When the two inhibitors were transfected, they did not reverse the CSE-induced insulin sensitivity reduced (Figure 6E). We also measured the miR-153 levels in CS-exposed mice. In the hippocampal tissue of mice, CS led to high miR-153 expression (Figure 6F). These results indicate that miR-153 affects the PI3K/GSK3β/p-Tau pathway by modulating its target, the PI3K regulatory subunit PIK3R1.

## 4. Discussion

CS contains toxic chemicals that can affect the normal functioning of cells and tissues, including the brain. There is currently controversy over the relationship between cigarette smoking and Alzheimer’s and Parkinson’s diseases. However, there are still many studies supporting cigarette smoking as a risk factor for Alzheimer’s and Parkinson’s diseases. For example, Kaaren G. Wooten et al. found that smoking is a risk factor for Alzheimer’s disease and related dementia (ADRD), while Roch A. et al. found that controlling smoking is one of the ways to improve Alzheimer’s disease and related dementia (ADRD) [31,32]. The three dementia risk factors identified in the 2019 Global Dementia Prevalence Estimate and the 2050 Predicted Prevalence Study include high body mass index, high abdominal blood sugar, and smoking [33]. Although studies have shown that nicotine has a certain therapeutic effect on cognitive and memory abilities, cigarette smoke, as a complex mixed exposure, contains harmful substances such as ROS, arsenic, cadmium, and benzopyrene in addition to nicotine, which may cause oxidative stress, inflammation, and even directly cause brain damage through the blood–brain barrier [34,35]. The mechanism of the damage caused by cigarettes to the nervous system is not yet clear, but cigarette-induced insulin resistance has been reported in many studies, including studies on cigarette-induced peripheral insulin resistance [36,37]. Studies have shown that nicotine may cause metabolic disorders, increased blood sugar levels and disorders of glucose homeostasis by causing genetic variations in the nicotinic acetylcholine receptor, and then induce insulin resistance [38]. Thus, it is worth exploring whether cigarettes may cause neurological damage by causing peripheral and central insulin resistance.

Previous studies discovered that cigarette smoking causes neuronal oxidative stress, DNA damage, mitochondrial damage, and autophagy in brain cells [39,40]. Research from the China National Tobacco Quality Supervision and Test Center revealed lower mitochondrial DNA (mtDNA) copy numbers in the peripheral blood of healthy smokers than in non-smokers; these effects were mediated by autophagy. Sebastiano et al. [41] determined that, when CD-1 mice were exposed to CS for four weeks, lipid peroxides, DNA damage, and apoptosis were present in the brain. They also demonstrated that CS exposure elevated the microtubule protein Tau; our study found similar effects for C57BL/6 mice.

There are numerous animal models of CS exposure, some focusing on the effects of prenatal CS exposure on the brains of offspring [42] and others on the impairment of cognitive function in adulthood from neonatal CS exposure. After adult male Sprague Dawley rats were exposed to CS for 3 h per day for three weeks, there were altered levels of glial fibrillary acidic protein and elevated numbers of apoptotic cells. These studies, with short CS exposures, found no appreciable deletion of neuronal pyramidal cells in pathological sections. Therefore, the present study selected six-month-old mice for six-month exposures to CS. According to the results of H&E, Nissl, and NeuN immunohistochemistry, long-term CS exposure caused a loss of pyramidal or granule neurons in the hippocampus of aged mice (12 months old). However, there are some results in the current research on neuronal damage caused by cigarette smoke exposure that differ from our findings. The study by Dobric A and others [43] found that 6 months of cigarette smoke exposure (419 mg/m^3^ TPM) impairs the working memory, decreases microglial numbers, and increases microglial activation in the hippocampus, but does not show an impact on neurons. The animal model used in this study was BALB/c mice, and the cigarettes used were Winfield Red Cigarettes. This suggests that the different proportions of substances in cigarettes and differences in animal models may affect the manifestations of brain damage caused by cigarette smoke. However, despite these differences, the impact of long-term cigarette smoke exposure on cognition and the nervous system is consistent.

In exploring the mechanisms of CS-induced neurotoxicity, we discovered that CS exposure altered the PI3K/GSK3β signaling pathway, diminishing insulin sensitivity and increasing Tau hyperphosphorylation. The PI3K/GSK3β signaling pathway is the classical insulin signaling pathway activated via insulin/insulin-like growth factor [44]. Insulin resistance in the brain is a condition in which brain cells become less responsive to the effects of insulin, leading to a decline in cognitive function and an increased risk of neurodegeneration [4]. Insulin signaling begins with the activation of the insulin receptor by insulin. Subsequently, PI3K phosphorylates PIP2 to PIP3, activating the AKT signaling pathway and negatively regulating GSK3β [45]. PI3K is a heterodimer composed of regulatory and catalytic subunits [46]. Of particular relevance to AD, some GSK-3β targets, including Aβ peptide and Tau protein, are pathological hallmarks of AD [47]. We noted that the CSE exposure of SH-SY5Y cells lowered PI3KR1. 

Although the mechanism by which CS influences the brain insulin signaling pathway and neurotoxicity is unknown, miRNA dysregulation is a potential mode of action. We performed a target gene search for 3′-UTR regions against an miRNA database and found that miR-153-3p targeted the PI3KR1 3′-UTR. Paraquat increases the expression of brain miR-153 and induces dopaminergic neurotoxicity [48]. In cerebrospinal fluid exosomes of PD and AD patients, miR-153 is overexpressed, and miR-153 expression is elevated in primary islets of diabetic db/db mice [49].

Our results indicate that, in the brain, miR-153 is involved in CS-induced neuron insulin resistance and in a disorder of the PI3K/GSK3β/Tau pathway. The exposure of mice to CS caused memory and cognitive impairment and a deletion of pyramidal and granule cells in the CA1 and DG regions of the hippocampus. Our results suggest that long-term exposure to 200 mg/m^3^ and 300 mg/m^3^ doses of cigarette smoke can cause significant memory damage and pathological changes, suggesting that long-term moderate or severe smoking may cause neurotoxicity in the population. The results provide a better understanding of the neurotoxicity caused by exposure to CS and may be useful for the development of treatment and/or prevention strategies for neurodegenerative diseases. Individuals must be aware of the association between chronic moderate to severe smoking and increased neuropathological damage, and seek assistance in quitting smoking when necessary.

However, this study contains some limitations. Compared to SH-SY5Y cells, primary neuronal cultures could provide additional insights into the physiological relevance of our findings. In future studies, we will use primary neuronal cultures or hippocampal slices to validate the physiological implications of cigarette smoke exposure on neuronal insulin signaling. In addition, this study only included male mice, which caused certain limitations in fully elucidating the neural damage caused by smoking.

## Figures and Tables

**Table 1:** miRNA primer sequences.

*miR-153*	*5′-GCUAAAUUCACAGCGCUAUGCAA-3′*
	*3′-CUAGUGAAAACACUGAUACGUU-5′*
*miR-17*	*5′-GAGGCAAGGUUAUAUGCACUUUC-3′*
	*3′-GAUGGACGUGACAUUCGUGAAAC-5′*
*miR-128*	*5′-GACCCAGACACAUCGCACUGUGG-3′*
	*3′-UUUCUCUGGCCAA-GUGACACU-5′*
*miR-590*	*5′-AAAUGUACCUUCAGAAUAAGCUUC-3′*
	*3′-GACGUGAAAAUACUUAUUCGAG-5′*
*U6*	*5′-CGCTTCGGCAGCACATATACTAAAATTGGAAC-3′*
	*3′-CGTTCCTACTGTGCGTTTAAGCACTTCG-5′*

## Data Availability

The data that support the findings of this study are available from the corresponding author, Huanwen Tang, upon reasonable request.

## Supplementary Materials

The following supporting information can be downloaded at: https://www.mdpi.com/article/10.3390/toxics11120969/s1, Figure S1. The effects of CS concentrations on body weight. Figure S2. Effects of various concentrations of CS on brain levels of Nissl and NeuN. Figure S3. CS induces pyramidal cell changes in the CA3 and DG regions in the hippocampus of mice.
