# Supplementary material for: miR-153-3p via PIK3R1 Is Involved in Cigarette Smoke-Induced Neurotoxicity in the Brain
Source: Toxics. 2023 Nov 30;11(12):969. doi: 10.3390/toxics11120969 (PMC10747656; doi:10.3390/toxics11120969)
Supplement: Supplementary file 1 [file toxics-11-00969-s001.zip › toxics-2685770-supplementary.pdf]

Supplementary Figures

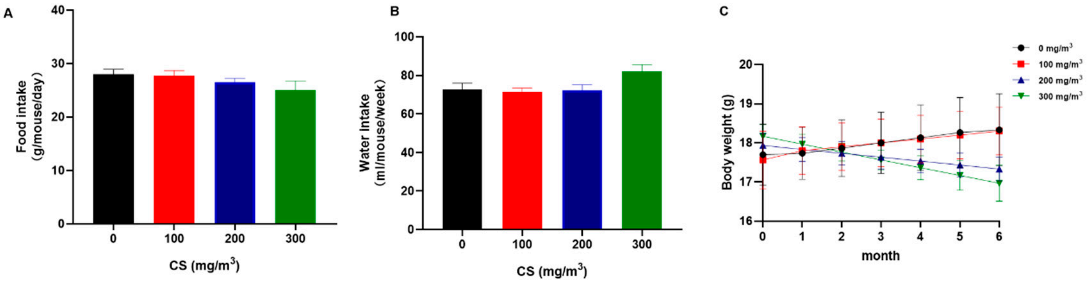

Figure S1. The effects of CS concentrations on body weight.

C57BL/6 mice were exposed to 0, 100, 200, or 300 mg/m^3 total particulate matter (TPM) CS for six months. Food intake and water intake were recorded daily and averaged over 6 months (A) Food intake. (B) Water intake. Weights were recorded weekly, and the average weights were calculated monthly. The weight change curve was plotted by month. (C) Body weights.

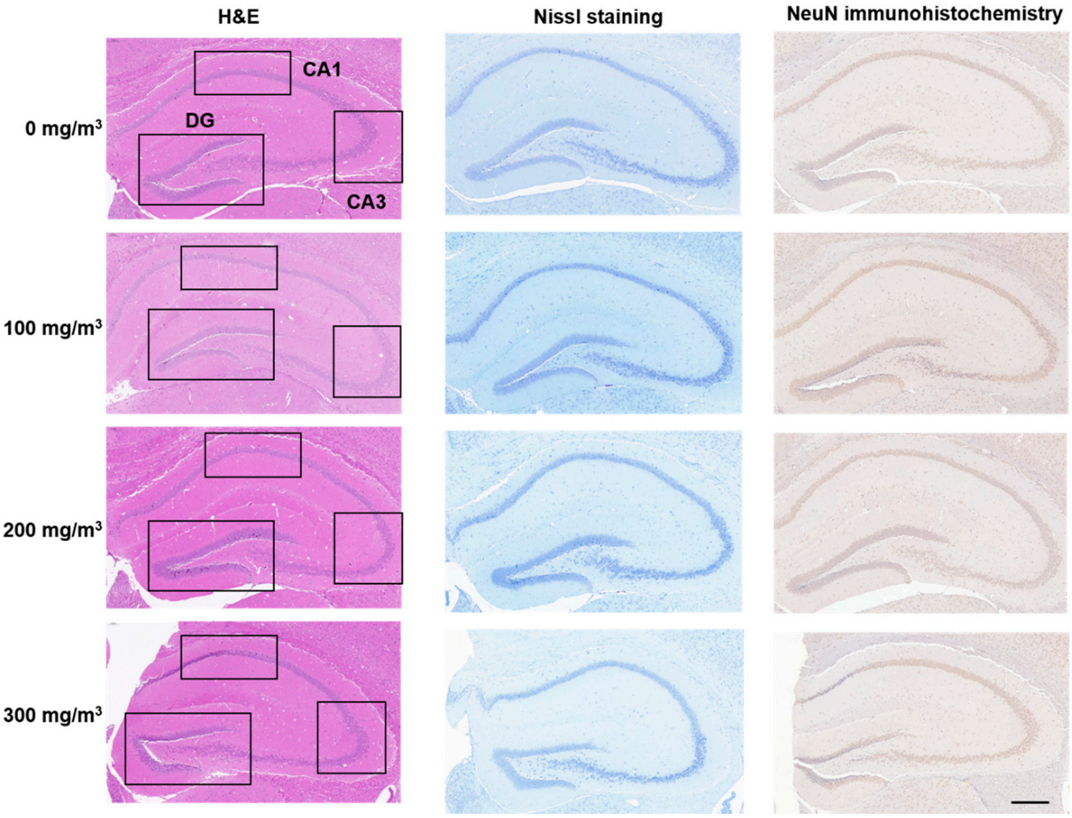

Figure S2. Effects of various concentrations of CS on brain levels of Nissl and NeuN.

C57BL/6 mice were exposed to 0, 100, 200, or 300 mg/m^3 total particulate matter (TPM) CS for six months. Histological examinations, and Nissl staining and NeuN immunohistochemistry of mouse brain sections were performed to evaluate brain damage. Representative photomicrograph of H&E staining, Nissl staining, and NeuN immunohistochemistry in the hippocampus. Scale bar =500 μm.

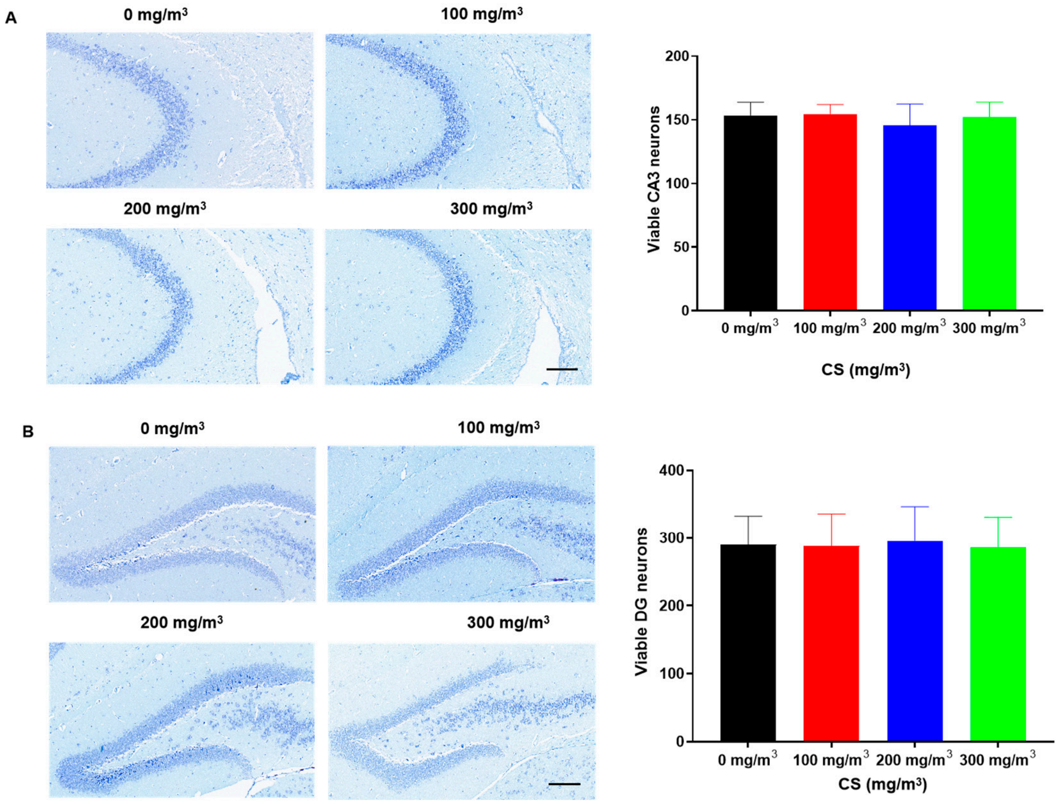

Figure S3. CS induces pyramidal cell changes in the CA3 and DG regions in the hippocampus of mice.

Representative photomicrograph of Nissl staining in the hippocampus. Scale bar =50 μm. （A-B）No significant differences were seen in the effects of CS exposure on pyramidal neurons in the CA3 and DG regions of mice. All data were presented as means ± SD for experiments conducted in triplicate. (n=6 for each

group).
